# An acoustic model of speech dysprosody in patients with Parkinson's disease

**DOI:** 10.3389/fnhum.2025.1566274

**Published:** 2025-04-28

**Authors:** Fredrik Nylén

**Affiliations:** Department of Clinical Science, Faculty of Medicine, Umeå University, Umeå, Västerbotten, Sweden

**Keywords:** automatic acoustic assessment, dysprosody, Parkinson's disease, dysarthria, prosody

## Abstract

**Purpose:**

This study aimed to determine the acoustic properties most indicative of dysprosody severity in patients with Parkinson's disease using an automated acoustic assessment procedure.

**Method:**

A total of 108 read speech recordings of 68 speakers with PD (45 male, 23 female, aged 65.0 ± 9.8 yea*rs*) were made with active levodopa treatment. A total of 40 of the patients were additionally recorded without levodopa treatment to increase the range of dysprosody severity in the sample. Four human clinical experts independently assessed the patients' recordings in terms of dysprosody severity. Separately, a speech processing pipeline extracted the acoustic properties of prosodic relevance from automatically identified portions of speech used as utterance proxies. Five machine learning models were trained on 75% of speech portions and the perceptual evaluations of the speaker's dysprosody severity in a 10-fold cross-validation procedure. They were evaluated regarding their ability to predict the perceptual assessments of recordings excluded during training. The models' performances were assessed by their ability to accurately predict clinical experts' dysprosody severity assessments.

**Results:**

The acoustic predictors of importance spanned several acoustic domains of prosodic relevance, with the variability in *f*_*o*_ change between intonational turning points and the average first Mel-frequency cepstral coefficient at these points being the two top predictors. While predominant in the literature, variability in utterance-wide *f*_*o*_ was *fo*und to be only the fifth strongest predictor.

**Conclusion:**

Human expert raters' assessments of dysprosody can be approximated by the automated procedure, affording application in clinical settings where an experienced expert is unavailable. Variability in pitch does not adequately describe the level of dysprosody due to Parkinson's disease.

## 1 Introduction

Dysprosody is a well-attested symptom of Parkinson's disease (Schlenck et al., 1993) and is discussed in the literature as an “impaired melody of speech”, speaking monotony in pitch or loudness (“monopitch” and “monoloudness”, respectively), “hypophonia”, or an “altered rate of speech” (Sidtis et al., 2006). Dysprosody is an early-onset symptom of the disease (Schlenck et al., 1993) and a prominent factor behind reduced speech intelligibility (Watson and Schlauch, 2008; Klopfenstein, 2009; Feenaughty et al., 2014; Martens et al., 2015) and communicative efficiency (Martens et al., 2011). Dysprosody is most often discussed in connection with dysarthrias and predominately in connection with Parkinson's disease (PD) and Huntington's disease (Rusz et al., 2014). Effects on expressive dysprosody have, however, also been observed following lesions in the caudate nucleus, the globus pallidus, and the putamen (Sidtis and Sidtis, 2003), in case reports of left hemiparesis and right hemisphere tumo*rs* (Sidtis, 1984), and in ~2.7% of patients with epileptic seizures (Peters et al., 2011). When occurring as a component of apraxia of speech (Ballard et al., 2016), symptoms have been observed to be alleviated by neurobehavioral treatment (Ballard et al., 2010). There is, therefore, a great need to further our unde*rs*tanding of dysprosody-causing symptoms of diseases and develop a robust assessment method to guide diagnosis and management of treatments across several neurological conditions. However, while widely attested and often discussed in reports of speech effects of PD and other neurological diseases, there is currently no objective measure of dysprosody by which the impact of treatment may be assessed (Steurer et al., 2022).

One barrier to developing acoustic assessment methods for dysprosody originates in the complex nature of prosody itself (Terken and Hermes, 2000; Sidtis and Sidtis, 2003; Ladd and Arvaniti, 2022). A recent review by Fumel et al. (2024) highlighted nine aspects (*f*_*o*_, the variability in *f*_*o*_, intensity, intensity variability, speech rate, articulation rate, pause duration, and proportion of pauses during speaking) that have been the focus of previous research on dysprosody. Utterance-wide variability in *f*_*o*_ is the most often used proxy measure for dysprosody, in which reduced *f*_*o*_ excu*rs*ions have been a consistent finding in patients with Parkinson's disease compared to control speakers (Bocklet et al., 2011; MacPherson et al., 2011; Skodda, 2011; Feenaughty et al., 2014; Thies et al., 2019; Frota et al., 2021) across many languages (Fumel et al., 2024). As noted by Fumel et al. (2024), however, variability in *f*_*o*_ does not afford reliable interpretation in terms of dysprosody or severity since modulation of *f*_*o*_ is also linked with the perception of liveliness, emotional expressions, or speaker gender (Traunmüller and Eriksson, 1995; Avery and Liss, 1996; Martinho et al., 2024; Nylén et al., 2024). Dysprosody as a term should, however, be used only to describe specifically the effects that may reduce speech intelligibility, and other indexical properties should not be considered in the assessment (Fumel et al., 2024). Utterance-wide variation in speech intensity is the second most common proxy measure of dysprosody, for which Fumel et al. noted some evidence of a systematic reduction in patients with PD compared to control speakers in their review. Still, the effect was less consistent than the corresponding effect on *f*_*o*_ variability. Some dopaminergic treatments, e.g., levodopa administration or deep brain stimulation (DBS) in the subthalamic nucleus (STN), may alleviate the adve*rs*e effects of PD on *f*_*o*_ variability (Lundgren et al., 2011; Skodda, 2011; Karlsson et al., 2013; Thies et al., 2024). Possibly, good alleviation may require elevated treatment levels to have beneficial effects (Bobin et al., 2024). The beneficial effects are, however, not unive*rs*ally observed across dopaminergic treatments. DBS in the posterior subthalamic area may, in contrast, have no beneficial effect on PD speakers' ability to modulate *f*_*o*_ or intensity on the global level (Lundgren et al., 2011; Karlsson et al., 2013) or speech intelligibility (Johansson et al., 2014; Sandström et al., 2015).

Dysprosody can also manifest in speech rhythm through speech rate and articulation effects. According to the review by Fumel et al. (2024), these effects are more negligible and are less systematically observed across languages. As noted by Liss et al. (2009), speech rhythm deviation can be used to correctly classify speakers into dysarthria types with 80% prediction accuracy when quantified using manually annotated syllable nuclei and onset and coda component relationships (Liss et al., 2009). Retained control over articulation rate and consonant/vowel relationships in speech motor tasks can be used to correctly identify speakers with PD among controls (Karlsson and Hartelius, 2019) and to predict dysarthria severity (Karlsson et al., 2020), but increasing age of the speaker also affects these properties (Karlsson and Hartelius, 2021), which makes them challenging to use as marke*rs* of disease progression. A reduced speech rate has only been reliably observed in American English and Dutch (Fumel et al., 2024). The frequency and length of pauses in speech were a much more systematic observation separating PD speakers from healthy controls in the meta-analysis by Fumel et al. than speech or articulation rate effects. Dopaminergic treatments using levodopa or DBS in the STN have been observed to alleviate, but not entirely extinguish, the speech and articulation rate effects (Ho et al., 2008; Karlsson et al., 2011; Knowles et al., 2024) and can be further amplified by DBS stimulation that is adjusted in real-time in response to bioelectrical signals from the patient (Cernera et al., 2024).

While observed with reasonable consistency, utterance-wide reductions in acoustic expressiveness due to PD, the effects are not large enough or observed with sufficient consistency for assessing dysprosody severity. Well-functional prosody is a well-explored field of linguistics, and analytical techniques have been used to provide a more detailed, time-aligned view of how PD affects speakers' prosodic ability. The autosegmental-metrical (AM) analysis, in which the realization and temporal alignment of language-specific intonational units (tones) and the strength of breaks (pauses) are categorized, has, in case reports, been used to observe a reduced concentration of prosodic tonal events due to PD. In contrast, the repertoire of tones used in communication has been observed to remain unaltered (Lowit et al., 2014). The AM framework has substantial descriptive value but does not provide direct insight that can be transferred to a measure of dysprosody in the speaker. Frota et al. (2021), however, recently proposed an extension to AM for Portuguese (P-ToBI) that deduces a prosodic index from the difference between the pitch accents and breaks that are produced by the PD patient compared to what would be expected in unimpaired speech. Thies et al. provided supporting evidence for this approach by showing that *f*_*o*_ peak in the syllable nuclei (the vowel) is lowered by PD (Thies et al., 2019). Manual multi-tier annotation of utterance, pitch accents, and break indices before analysis (Thies et al., 2019; Frota et al., 2021). The most reliable models of dysprosody severity due to dysarthria have shown an accuracy of 62.2–73.9%, depending on the model type, when trained on a set of intonation (*f*_*o*_) and rhythmic properties extracted after the manual annotation of the utterance (Hernandez et al., 2020). Automatic segmental and syllable annotation procedures have been proposed. Still, they are challenged by syllable boundaries, to which tonal events are aligned, being more readily perceived as a cognitive construct with varying definitions (Vitale et al., 2024) than units that can unequivocally be segmented in recordings of fluent speech (Warren et al., 1996; Reetzke et al., 2021). Therefore, the manual work required to perform the analysis offe*rs* a barrier to adoption beyond use in research for these analytical techniques. A less laborious work is required to find the stress pattern index (Tykalová et al., 2013), defined as $1+ln\left(\frac{f_{o_{\max}}}{f_{o_{\min}}}\right)$ ) and the syllabic prosody index (Tavi and Werner, 2020) defined as $\frac{f_{o}\sqrt{d}}{\sqrt{E_{f<1kHz}}}$ (where *f*_*o*_ is the fundamental frequency, *E* the speech signal energy, and *d* is the duration) of words and syllables, respectively. These measures have, however, been evaluated in small samples of participants only (36 PD patients and controls) and only in terms of their ability to separate speaker groups. An evaluation of the affordance to accurately predict levels of dysprosody based on these metrics has not been attempted.

It should be observed that the properties discussed concerning dysprosody assessment in PD and other neurological diseases are only a subset of the features supporting appropriate prosody perception. Recent studies (Roessig et al., 2022; Arvaniti et al., 2024; Hu and Arvaniti, 2024) have highlighted additional cues that warrant renewed attention concerning the perception of prosodic entities. Vowels' spectral balance (spectral tilt) has long been attested to contribute to the perception of prominence in many languages (Sluijter and van Heuven, 1996; Heldner, 2003; Crosswhite, 2003) with a relative predictive strength rivaling the strongest cue (duration) (Sluijter and van Heuven, 1996; Heldner, 2003). Several proposals of how spectral tilt or spectral balance should be quantified have been proposed, and the Spectral Energy Ratio (SER) between a lower frequency band (0–1 kHz) and a higher frequency band (1–5 kHz) (Murphy et al., 2008) provides an intuitive base approximation of the tilt of the spectrum. However, the first Mel frequency cepstral coefficient (C1) and components of a first or sixth-order polynomial fitted to the logarithmic magnitude spectrum (SLF and SLF6D) have been shown to provide more robust quantifications (Kakouros et al., 2018). The level relation of the first and second, or first and third (Okobi, 2006), harmonic, both directly measured from the speech signal (L_2_-L_1_ and L_3_-L_1_) (Kakouros et al., 2018) and with correction (Iseli et al., 2007) for the effect of neighboring formants (corrected L_2_-L_1_ and L_3_-L_1_) (Hu and Arvaniti, 2024), have also been proposed to be potent cues.

A structure hinged on acoustic parameters is required to achieve an automatized framework for assessing dysprosody. Here, the outcome of observations from two different developments is fused to explore ways to circumvent the barrier to automatic assessment of dysprosody introduced by requirements for reliable syllable or segment isolation. First, it is observed that rhythmic structure can efficiently be extracted from the overall modulation of the speech signal (Leong et al., 2014) and that this information can be used to separate dysarthria types (LeGendre et al., 2009; Liss et al., 2010). While previous studies have explored envelope modulation to deduce rhythmic structure, it is proposed here that the timing of prosodically significant tonal events may provide an indirect cue to rhythmic properties by hinting at the approximate timing of the associated syllable.

Second, some previous studies have been directed toward stylizing a computed *f*_*o*_ curve into a more efficient and appropriate representation of the intonational structure, with microvariations removed. Taylor (1994) applied a *rise, fall*, and *connection element* classification scheme to a two-step median smoothed *f*_*o*_ and associated assigned elements with time and amplitude scaling facto*rs* (parameters) to deduce a representation of intonation that could replace the AM annotation in an automatic procedure. The degree of change in *f*_*o*_ was observed to provide a cue to the presence of a pitch accent. The subsequent Tilt model (Taylor, 2000) expanded the analysis by associating a rise amplitude, rise duration, fall amplitude, and fall duration with each element to derive a representation that could relatively faithfully reproduce manual annotations in analyzing a smoothed *f*_*o*_ curve in synthesis (Taylor, 2000). The reliance on a precomputed *f*_*o*_ with manual adjustments made to the speaker is a disadvantage to this approach when attempting automatic modeling.

**Figure 1:**
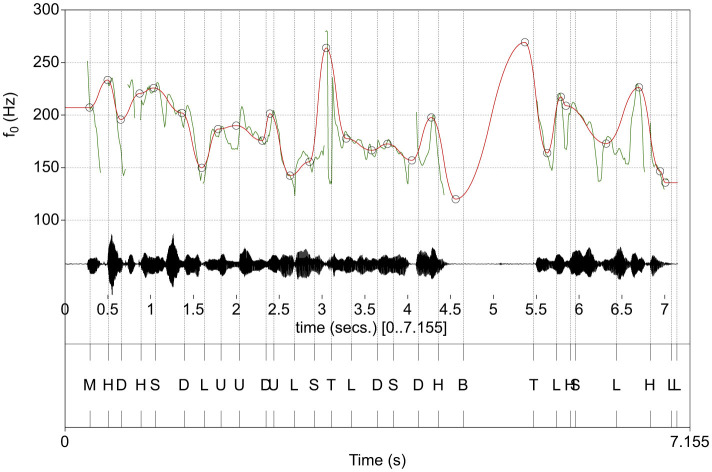
Example automatic INTSINT annotation (**bottom panel**) along with the audio waveform (**middle panel**) and computed *f*_*o*_ curve (**top panel, green**). The approximation of the macro-prosodic structure of the utterance estimated by the Momel algorithm is visualized is overlayed on the original *f*_*o*_ curve (**top panel, red**). Momel target points (MPTs) are marked with circles.

In an alternative approach *Modeling melody* (Momel) algorithm (Hirst, 2005) extracts, the *f*_*o*_ contour in a two-step procedure, where the first quartile (q_1_) of the distribution of *f*_*o*_ values obtained using very wide search space (typically 60–750 Hz) is used to derive the *f*_*o*_ curve forming the basis for subsequent computations within the search space of 0.75q_1_ Hz to 1.5 octaves above q_1_. This two-step process is proposed to reduce the need for age and sex adjustment of parameters when deriving the *f*_*o*_ curve. The *f*_*o*_ curve is then separated into one quadratic spline function representation aimed at capturing the macro prosodic representation and a similar micro prosodic representation, which is not considered further here. The target points (Momel target points, MTPs) in the macro prosodic quadratic spline function are defined as (time, frequency) points that define significant tonal events in the utterance. The International Transcription System for Intonation (INTSINT) establishes a series of annotations of an intonational curve into (T)op, (H)igher (local maximum), (U)ppstepped, (S)ame, (M)id, (D)ownstepped, (L)ower (local minimum), and (B)ottom level. A parameter *key* is obtained by stepwise search originating from the mean *f*_*o*_, and a *range* parameter is obtained in the 0.5–2.5 octave range. After defining the predicted *f*_*o*_ as an MTP by their INTSINT label as $T=key\sqrt{2^{span}}$, *M* = *key*, $B=\frac{key}{\sqrt{2^{span}}}$. The local maximum/minimum levels (H and L) model an *f*_*o*_ at the (log scaled) midpoint between the preceding MTPs and the T and B levels, respectively. Similarly, the up and downstepped levels (U and D) represent a point a quarter of the log-scaled distance between the previous MTPs and the T and B levels, respectively (Hirst, 2011). See Figure 1 for an illustration of the Momel and INTSINT automatic annotation output. The Momel and INTSINT annotation procedures have been given a canonical computer implementation (Hirst, 2007) and have been applied to describe intonation in relation to temporal events in several languages (Hirst and di Cristo, 1998; Véronis et al., 1998; Hirst et al., 2007; Chentir, 2009; Hirst, 2013; Celeste and Reis, 2021). The procedure has further been shown to reproduce human perception of tonal events with high accuracy (Hirst, 2011). If perceptually reliable, the MTPs could also serve as hints to prosodically relevant syllables and the rhythmic structure of speech. As the MTPs are defined in time and frequency, it is possible to associate intensity and spectral tilt measures with a time window surrounding the MPT to provide an augmented representation of prosody. Recent developments in vocal activity detection (Yin et al., 2018; Bredin et al., 2020; Bullock et al., 2020; Cristia et al., 2021) further suggest that units of speech approximating utterances could be automatically extracted from a speech recording prior to prosodic analysis in an unsupervised manner. When automatic assessments of all approximate utterances are combined, an assessment of dysprosody severity in the speaker may likely be deduced, and the combination of procedures into an analytical pipeline suggests a path toward a fully automated dysprosody assessment procedure.

The current study aimed to describe and evaluate a fully automated pipeline for the evaluation of dysprosody. Previous efforts to assess dysprosody have not had a solid base obtained from the perception of humans for evaluating the prediction accuracy of methods available to them, which is essential for a machine learning approach. Perceptual evaluations of dysprosody made by four speech and language pathologists with extensive experience in perceptual dysarthria assessment were used as ground truth for model training and evaluation. Four different machine learning model types with differing strengths and primary design aims were used to ensure that substantial contributions to our unde*rs*tanding of the perception of dysprosody could be represented regardless of the model's characteristics. Furthermore, the possibility of concentrating each model's strengths into an overall best-performing ensemble model was explored. The secondary aim was to describe the acoustic properties most indicative of increased dysprosody severity. A comparison of the predictive accuracy of the best-performing model with one with only one predictor, variability in *f*_*o*_ across an utterance, is also made.

## 2 Method

### 2.1 Perceptual evaluation of dysprosody

Audio recordings of 68 speakers with PD (45 male and 23 female) aged 65.0 ± 9.8 yea*rs*, and with an average Hoehn & Yahr (H&Y) (Hoehn and Yahr, 1967) rating of 2.42 ± 0.57, and an average Unified Parkinson's Disease Rating Scale motor score [UPDRS part III (Goetz et al., 2008)] of 33 ± 12 were included in this study. The participants were asked to read an 89-word text, in which statements, interrogations, assertions, and instances of role changes are included and is the standard text used in dysarthria assessment in which dysprosody is an assessed component [“Ett svårt fall” (“A difficult case”); Eklund et al. (2014)]. The included recordings were made while on L-dopa medication. To increase the range of speech impairment levels in the study, recordings where the patient was off levodopa medication were also included when available (*n* = 40). Consequently, the total set of read speech recordings analyzed was 108. All recorded speeches were made in a quiet room at either a 48 kHz (*n* = 79) or 44.1 kHz (*n* = 29) sampling rate using either a Sennheiser HSp 4 and an MZA 900 P phantom adapter or a Marantz PMD 660 digital audio flash recorder.

Four expert speech-language pathologists (SLPs) with extensive (>20 yea*rs*) experience in the assessment of dysarthria assessed all (108) readings of the standard text individually in four domains (“Articulation”, “Voice”, “Resonance”, “Prosody”, and “Overall impression”). A perceptual rating scale with four levels of deviant production (“No deviation”, “Mild”, “Moderate”, or “Severe” deviation) was used in the perceptual assessment. As previously described (Karlsson et al., 2020), the moderate and severe categories were subsequently merged into a “Moderate to severe” category to support model training due to too few ratings of severe deviation. Spearman's correlations between the perceived level of deviation in prosody compared with other rated dimensions were strong for “Overall impression” (*r*_*s*_ = 0.73) and “Articulation” (*r*_*s*_ = 0.65), moderate for “Voice” (*r*_*s*_ = 0.55), and weak for “Resonance” (*r*_*s*_ = 0.25). An initial consensus training session in which four readings were rated and discussed for consensus was performed before the perceptual assessment to strengthen inter-rater reliability. Laptops and Sennheiser HD 212Pro headsets were used in the perceptual evaluation.

### 2.2 Speech signal processing

The audio recordings were segmented into vocal activities approximating read speech sentences using overlap-aware speech detection (Bredin et al., 2020; Bullock et al., 2020). The identified portions of speech acts were then submitted to Momel & INTSINT processing, in which the *f*_*o*_ tracks (using a 10 ms analysis window), utterance *f*_*o*_ key, and *f*_*o*_ range were automatically identified, and MTPs were derived from the *f*_*o*_ track. INTSINT annotations were then assigned to each MPT on the macro-prosodic intonational structure. The entire utterance and each MPT were then provided with acoustic quantifications presented in Table 1 using the analysis procedure presented in Supplementary material A.

**Table 1:** Descriptions of quantifications of each automatically extracted utterance.

**Domain**	**Description of measure**	**Number of predictors per utterance**
Time (s)	The time points relative to the start of the utterance associated with MTPs (in s)^†^	6
	The time distance from the previous MTPs (in s)	6
	The duration of the utterance (in s)	1
	The concentration of MTPs in an utterance (MTP/s)	1
	Duration of the utterance	1
*f_*o*_* (Hz)	The Momel *f_*o*_* associated with the MTPs^†^	6
	The change in Momel *f_*o*_* from the previous MTPs^†^	6
	The *f_*o*_* key of the utterance	1
	The range of the Momel *f_*o*_* within the utterance	1
	The minimum of the Momel *f_*o*_* within the utterance	1
	The maximum of the Momel *f_*o*_* within the utterance	1
Intensity (dB)	The speech signal intensity associated with the MTPs^†^	6
	The change in speech signal intensity from the previous MTPs^†^	6
	The *f_*o*_* key of the utterance	1
	The range of speech signal intensities within the utterance	1
	The minimum speech signal intensity within the utterance	1
	The maximum speech signal intensity within the utterance	1
Spectral tilt	The spectral energy ratio (in dB) between 0–1 kHz and 1–5 kHz at the MTPs	7
	The spectral energy ratio (in dB) between 0–1 kHz and 1–5 kHz from the previous MTPs^†^	7
	The L_2_-L_1_ (in dB) associated with the MTPs, both with and without correction^‡^ for the effect of nearby formants^†^	12
	The L_3_-L_1_ (in dB) associated with the MTPs, both with and without correction^‡^ for the effect of nearby formants^†^	12
	The change in L_2_-L_1_ (in dB) from the previous MTPs, both with and without correction^‡^ for the effect of nearby formants^†^	12
	The L_3_-L_1_ (in dB) associated with the MTPs^†^, both with and without correction^‡^ for the effect of nearby formants	12
	The first Mel-frequency cepstral coefficient at the MTPs^†^	6
	The change in the first Mel-frequency cepstral coefficient from the previous MTPs^†^	6
	The slope of a fi*rs*t order polynomial to the short-term logarithmic magnitude spectrum at the MTPs^†^	6
	The change in slope of a fi*rs*t order polynomial to the short-term logarithmic magnitude spectrum from the previous MTPs^†^	6
	The coefficients of a sixth order polynomial to the short-term logarithmic magnitude spectrum at the MTPs^†^	36
	The change in coefficients of a sixth order polynomial to the short-term logarithmic magnitude spectrum from the previous MTPs^†^	36
Total number of predictors extracted per utterance		205

^†^Summary statistics extracted for each utterance: minimum, maximum, mean, standard deviation, coefficient of variability, and inter-quartal range.

^‡^The correction was computed using the method presented by Iseli et al. (2006) and with formant bandwidths estimated using the method proposed by Hawks and Miller (1995).

### 2.3 Machine learning

The ability of the quantifications of the prosody in the automatically extracted utterances to serve as predictors of human experts' ratings of dysprosody (“No deviation,” “Mild,” and “Moderate to Severe deviation”) was evaluated in a cross-validation procedure. Five classification models with varying properties were selected for evaluation to explore their combined use in support of the study's aims to (1) develop and evaluate a model and analysis pipeline that could facilitate an automatic assessment of dysprosody and (2) determine which acoustic properties provide the best support for classifying dysprosody severity. The polynomial support vector machine (SVM) model maximizes the distance between classes in a multidimensional space and has been used to identify both neurological diseases (Haq et al., 2019; Lahmiri and Shmuel, 2019; Arora and Tsanas, 2021) and other diseases based on voice samples (Vouzouneraki et al., 2024). While primarily considered for binary classification tasks, it has been extended to predict multiple classes and has been applied, for instance, to the prediction of vocal expression of emotion (Shahbakhi et al., 2014).

The penalized ordinal regression optimizes the error with a tuned balance between penalty terms based on the summed squares and the norms of the coefficients, and it has been previously used in models of detailed motor deterioration of speech performance due to Parkinson's disease (Karlsson and Hartelius, 2019; Karlsson et al., 2020). Elastic-net regularization of the ordinal regression was chosen here as it has been shown to perform well in speech data with multicollinearity among predictors (Tomaschek et al., 2018). The Random Forest is an ensemble model-building procedure in which multiple decision trees are trained on random subsets of predictors and training data. Combined to make a single prediction, they are well-documented to provide good prediction accuracy (Noroozi et al., 2017; Arora and Tsanas, 2021; Vouzouneraki et al., 2024). The k nearest neighbors is a non-parametric model-building technique that conside*rs* proximity between samples and has been shown to perform very well in classification tasks for the speech of individuals with Parkinson's disease (Amato et al., 2021) and specifically for dysprosody (Majda-Zdancewicz et al., 2024) in a small sample of speakers with PD.

The five models were trained on a training data set consisting of acoustic predictors extracted from 75% of the recorded readings, matched with all expert raters' assessments of dysprosody in the patients' speech. The data were randomly divided into training (75%) and evaluation (25%) data sets, and the data were stratified to ensure a similar distribution of dysprosody severity in the two data sets.

The model parameters were tuned in a 10-fold cross-validation procedure. The models were optimized based on their ability to predict the perceptual assessments of utterances in the training data's 10th (holdout) fold. The model-tuning procedure used the mean logarithmic loss function to measure classification error. Highly correlated predictors (Spearman's *r* > 0.9; 43 predictors) were substituted for the predictor with the highest correlation with the outcome variable (the rating of dysprosody severity) before model training to produce better conditions for model training. The model tuning was performed using 1,000 parameter candidates for each hyperparameter (Table 2) that were spaced to maximize entropy in the distribution (Dupuy et al., 2015) with a variogram range of 2. The tuning procedure was repeated 10 times, each time with a different holdout portion of the data, and the final models were then constructed by averaging all 10 computed models of each type (support vector machines, penalized ordinal regression, k nearest neighbors, and Random Forest) to derive the final models. Furthermore, the models were combined into an ensemble model by model stacking and by weighing the predictions of each model relative to its strengths and weaknesses in prediction within the training data.

**Table 2:** Hyper-parameters tuned for each model in the 10-fold cross-validation procedure and the maximum and minimum hyper-parameter ranges in the tuning grid.

**Model name**	**Hyperparameter**	**Min**	**Max**
Polynomial support vector machines	The cost of predicting a sample inside of or on the wrong side of the margin	9.96 × 10^−4^	31.6
Penalized ordinal regression	The total amount of regularization	0.0	9.98 × 10^−1^
	The proportion of L1 and L2 penalization	6.7 × 10^−4^	9.99 × 10^−1^
Random forest	The number of trees	1	2,000
	The number of predictors sampled at each split	1	74
	The minimal number of data points at a node required for node split	2	40
k Nearest Neighbors	The number of neighbors considered	1	15
	The kernel function is used for weighing differences	Rectangular. Triangular. Epanechnikov. Bi-weight. Tri-weight. Cosine. Inve*rs*e. Gaussian. and Rank	
	The parameter used for calculating distance	

The importance of each variable in the most accurate model was computed using the feature importance ranking measure (FIRM) (Zien et al., 2009) procedure, which has the attractive property that it generalizes to the sum of the squared change in model output and, therefore, has a transparent interpretation independent of the model type investigated. The model training used a substantial set of acoustic predictors, 205 in total (Table 1). To reduce the risk of reporting a highly specialized ability of models to predict the data they were trained on and ensure generalizability, all model evaluations were performed on 25% of the data that were not part of model training but were set aside for model evaluation.

The final models were evaluated for their accuracy in predicting human raters' assessments of dysprosody in 25% of utterances not included during the training of the models. Similarly, the agreement among human raters on the most common assessment of the reading (consensus rating) was calculated. The consensus rating was chosen over assessment based on the level of inter-rater agreement to enhance the robustness of dysprosody assessment by collaborating clinical colleagues. Both human raters and computational models were tested on recordings for which they had not been informed of the actual outcome. The human raters were assessed using the same classification metrics as the trained machine learning models.

## 3 Results

The automatic extraction identified ~926 utterances from the 108 passage readings. The stratified sampling procedure aimed to create a comparable distribution of dysprosody severity levels across the training and test sets of utterances. A total of 684 utterances were assigned to the training set, while 242 were designated for the test set, which was used only at the evaluation stage.

**Figure 2:**
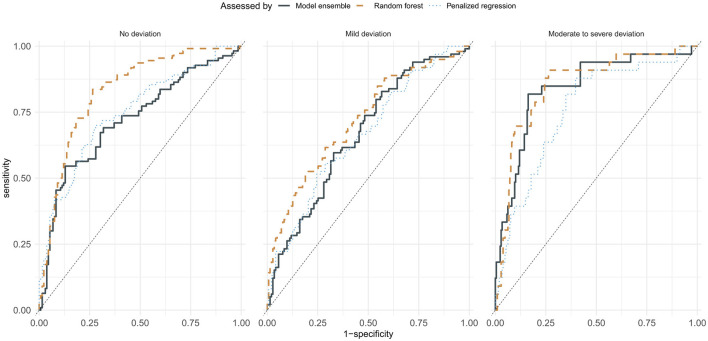
Receiver operating characteristic curves of random forest, the penalized ordinal regression, and ensemble models.

The performance of machine learning models in predicting the assessments of trained clinical professionals is presented in Table 3, along with the agreement of each of the four professionals' assessments with a majority rating for the utterance. Table 4 summarizes the inter-rater agreement between pai*rs* of raters. Human raters showed an average consensus (balanced accuracy in prediction) of 0.83 ± 0.04 (0.77–0.86) and an average *F* score of 0.73 ± 0.11 (0.56–0.80). The Support Vector Machines, Penalized ordinal regression, k Nearest Neighbors, and Random Forest models showed an average balanced accuracy in predicting unseen data of 0.66 ± 0.02 (0.63–0.68), with an average *F* score of 0.54 ± 0.02 (0.51–0.57). The best-performing model overall was the Random Forest model, with a balanced accuracy of 0.68 and an *F* score of 0.57; the Ensemble model training failed to produce a model that generalized well into the test data and showed performances that were lower than most original models, except for a strengthened positive predictive value of 0.69. The receiver operating characteristics (ROC) curves for the best-performing model (Random Forest), the model using the least number of predictors (penalized ordinal regression), and the model stack of all directly trained models (model ensemble) presented in Figure 2 indicate that the superior performance of the Random Forest model is achieved primarily by the model's ability to accurately predict cases rated as having “No deviation”.

**Table 3:** Agreement between the assessment of an individual rater, human expert, or acoustic model, and the true assessment of unobserved (testing) data.

**Truth**	**Prediction**	**Human raters**					**Acoustic models**					
		**Rater 1**	**Rater 2**	**Rater 3**	**Rater 4**	**Across all raters**	**Support vector machines**	**Penalized ordinal regression**	***k*** **nearest neighbors**	**Random forest**	**Ensemble**	**Model using variability in** ***f**_*o*_* **as the only predictor**
No deviation	No deviation	**26**	**25**	**31**	**20**	**102**	**74**	**77**	**83**	**80**	**23**	**14**
	Mild deviation	5	7	0	11	23	35	31	27	29	87	0
	Moderate to severe deviation	0	0	0	1	1	1	2	0	1	0	96
Mild deviation	No deviation	0	4	1	2	7	34	30	28	26	4	21
	Mild deviation	**19**	**18**	**24**	**11**	**72**	**54**	**59**	**63**	**63**	**94**	**0**
	Moderate to severe deviation	2	2	5	7	16	11	10	8	10	1	78
Moderate to severe deviation	No deviation	0	0	1	0	1	8	9	4	3	2	4
	Mild deviation	2	0	2	0	4	16	14	22	20	26	0
	Moderate to severe deviation	**4**	**6**	**3**	**3**	**16**	**9**	**10**	**7**	**10**	**5**	**28**
	Sensitivity	0.80	0.84	0.77	0.73	0.78	0.50	0.53	0.53	0.56	0.44	0.33
	Specificity	0.92	0.88	0.94	0.81	0.89	0.76	0.78	0.79	0.80	0.72	0.65
	Positive predictive value	0.80	0.78	0.75	0.56	0.71	0.53	0.56	0.58	0.59	0.69	–
	Negative predictive value	0.91	0.88	0.93	0.79	0.88	0.76	0.78	0.80	0.80	0.78	0.67
	Balanced accuracy	0.86	0.86	0.85	0.77	0.83	0.63	0.66	0.66	0.68	0.58	0.49
	*F*-score	0.80	0.80	0.75	0.56	0.73	0.51	0.54	0.54	0.57	0.40	0.20

The majority vote was considered the true outcome in human experts' assessments.

**Table 4:** Inter-rater agreement in dysprosody severity assessments among human raters.

**Compared ratings**	**% Agreement**	**Cohen's K**
Rater 1–Rater 2	69	0.47
Rater 1–Rater 3	62	0.35
Rater 1–Rater 4	44	0.18
Rater 2–Rater 3	66	0.40
Rater 2–Rater 4	46	0.22
Rater 3–Rater 4	43	0.15

**Figure 3:**
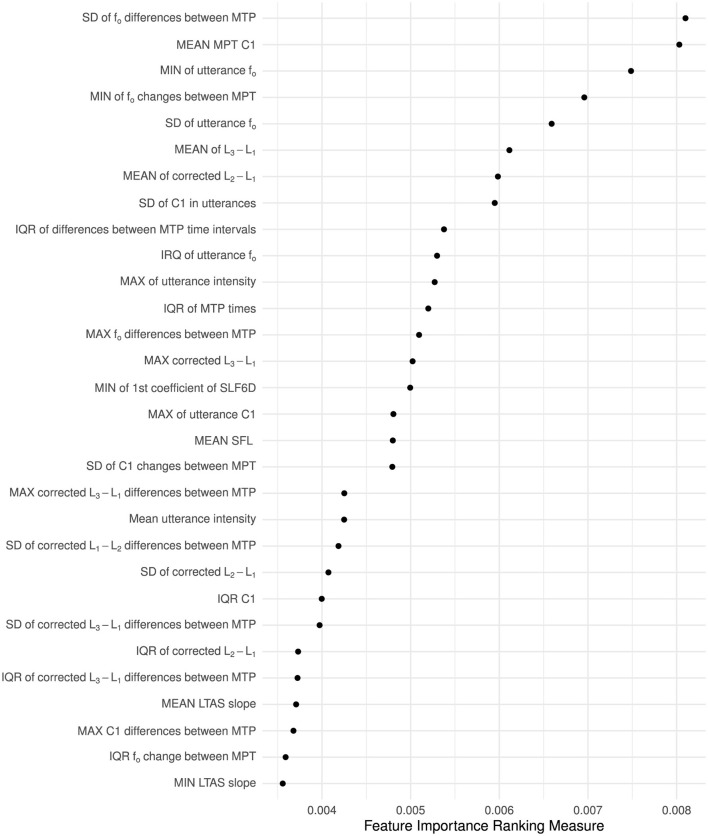
Variable importance of the 30 most important predictors extracted from MPT changes from one MPT to the next or across entire utterances. The importance of each variable was computed using the feature importance ranking measure (Zien et al., 2009) procedure. Notes: MTPs, Momel target points; LTAS, long-term average spectrum; C1, The first Mel-frequency cepstral coefficient; SLF, SLF6D, coefficients of a polynomial (with 1 and 6 components, respectively) fitted to the short-term logarithmic magnitude spectrum; MIN, MAX, MEAN, SD, and IQR indicate the summary statistic (minimum, maximum, mean, standard deviation, and interquartal range) applied to an utterance's quantifications to derive a single measure for each utterance. Measures described as “corrected” have spectral magnitude corrections (Iseli et al., 2007).

Figure 3 presents the FIRM (Zien et al., 2009) variable importance of the top 30 acoustic predictors in the best-performing Random Forest model. As a point of reference, an ordinal regression model in which variability in *f*_*o*_ was the only predictor of dysprosody severity showed a reduced balanced accuracy (0.49) and *F* score (0.20) compared to other models.

## 4 Discussion

Prosody is the language function that organizes the speech stream into manageable chunks for the listener to process, and failure to meet listeners' expectations is linked with reduced speech intelligibility. Prosody is inherently multidimensional in how it is signaled to the listener, and previous models aimed to detect neurogenic dysprosody severity have achieved 62.2–73.9% detection accuracy by incorporating information from intonation, rhythm, and pausing, information that was acquired through a manual annotation procedure. The requirement of a laborious and time-consuming transcription task preceding assessment presents a clear barrier to the clinical adoption of the assessment procedure. In this study, an automatic dysprosody assessment pipeline for speech utterance identification and pitch contour preprocessing was constructed and provided with a comprehensive quantification aimed at capturing aspects established to be prosodically important from the speech signal of an utterance. The complete pipeline was then assessed regarding its proficiency in assessing the dysprosody severity of patients with Parkinson's disease based on a recording of speech patients' reading, with no prior pre-processing. Five models were trained on the individual assessments of levels of dysprosody severity made by four clinical raters with extensive experience in assessing dysarthria and evaluated in terms of their ability to predict the consensus assessment of dysprosody severity among expert human raters on unobserved utterances.

The results suggest that the severity of dysprosody is not well described by single metrics, including the predominant proxy measure for dysprosody (utterance-wide variability in *f*_*o*_). Simpler bases for classification tended to result in strongly biased predictions that do not reflect human experts' ratings well, and no acoustic predictor showed an influence on the classification that was strong enough to serve as a proxy in determining dysprosody severity. A model of dysprosody assessment based on the utterance-wide variability of *f*_*o*_ alone showed a strong preference for classifying most samples (84%) as having “Moderate to severe deviation”. When using all predictors, the ensemble models Random Forest and penalized ordinal regression showed the best ability to identify utterances in the evaluation set that human experts had determined to have moderate to severe deviation in prosody. The support vector machine models failed to reach competitive levels of accuracy across all dysprosody severity levels. Overall, the Random Forest model achieved sensitivity, positive and negative predictive values, and an *F* score of predictions comparable to that of one rater (Rater 4) while not achieving similar sensitivity and balanced accuracy levels as the human rater. Overall, the acoustic models showed a lower sensitivity in their predictions than all human raters.

The result demonstrates that human clinical experts' assessment of dysprosody severity in Parkinson's disease can be partially modeled by a fully automatic speech processing pipeline in which utterances are automatically identified and that an intonation stylization can provide the scaffolding required for extracting acoustic cues. The developed models shed light on what constitutes a symptom of perceived dysprosody due to Parkinson's disease. While utterance-wide variability in *f*_*o*_ was not identified as a robust indication of the perceived level of dysprosody, the degree of variability, as well as the minimum of how much *f*_*o*_ changed from one MPT to the next, were identified as strong predictors. The modeling further highlighted that disregarding the first Mel spectrum coefficient and the level differences between the first and second, as well as the first and third harmonics, severely reduces the ability of an acoustic model to approximate human perception of dysprosody. The expert raters studied were not specifically experts in assessing dysprosody but were well-established experts in assessing dysarthria in a clinical setting, and the findings can, therefore, be transferred to a clinical setting.

Thus, previous reports in which dysprosody has been evaluated solely based on the proxy measure of the standard deviation of *f*_*o*_ are likely to have determined, in part, the level of liveliness (Traunmüller and Eriksson, 1995) in speech. Liveness is essential to our speech and likely contributes significantly to the experience of both parties in a communicative setting. However, utterance-wide variability in *f*_*o*_ alone does not ensure a retained linguistically functional intonation that adequately supports the transfer of information from the speaker to the listener. Instead, estimates of more local alterations in intonation, spectral balance, and intensity are used to distinguish portions of the speech signal of particular importance for the message from relatively less significant portions, providing a better model of clinical judgments of reduced prosodic functioning in patients with Parkinson's disease. Patients with Parkinson's disease have previously been observed to be reduced in their rapid regulation of phonation (Goberman et al., 2002; Goberman and Blomgren, 2008; Karlsson et al., 2012; Eklund et al., 2014; Tsuboi et al., 2014; Tanaka et al., 2015; Whitfield and Goberman, 2015), which may provide a partial explanation of the finding of less rapid local changes in *f*_*o*_ being significant predictors of clinically rated dysprosody specifically for patients with Parkinson's disease. While an explanation for the observations in terms of neurofunctional correlates cannot be offered to date, the connection with the subcortical structures, the globus pallidus, and the putamen (Sidtis and Sidtis, 2003) is congruent with an interpretation that failure to achieve tonal targets by pe*rs*ons with Parkinson's disease may be related to a failure to initiate an alteration of state in the phonatory musculature rather than an effect of muscular inability or fatigue or conflicting signaling in the direct, indirect, or hyper-direct pathways from the striatum to the cortex (Utter and Basso, 2008). This interpretation is, however, tentative and requires experimental support before being accepted.

Dysprosody is discussed here and in other parts of the literature as a single symptom. While discussed under a single term, dysprosody of a rated severity due to Parkinson's disease may differ from dysprosody caused by other neurological conditions (Sidtis, 1984). The automatic processing pipeline developed here does not presuppose a particular language or underlying disease causing dysprosody, but the relative importance of weights may likely be different for other diseases. Adjustments can, however, only be made with access to clinical raters with sufficient levels of experience and expertise. The procedure used in extracting acoustic parameters is made publicly available (Supplementary material A), and the procedures used for utterance segmentation and intonation modeling are widely available and well-documented (Hirst, 2007; Hirst et al., 2007; Origlia et al., 2013; Jadoul et al., 2018; Yin et al., 2018; Bredin et al., 2019; Bullock et al., 2020), which, when combined, removes any barrier to replication, language or disease estimates, adjustments in weights, and replication efforts in later research.

## 5 Conclusion

The perception of dysprosody can be approximated using an intonation stylization algorithm and an associated comprehensive acoustic assessment with no manually added temporal or tonal information. A performance in dysprosody assessment that approximates the abilities of clinical expert raters was achieved, which affords the transfer of a clinical assessment to remote situations where an experienced clinical expert is unavailable. The variation in pitch across an utterance, which is the most often used quantification of dysprosody in neurological disease, is not a reliable predictor of the level of dysprosody in patients with Parkinson's disease.

## Data Availability

All derived data supporting the conclusions of this article will be made available by the authors, without undue reservation. The speech recordings are sensitive personal identifiable information under national law and cannot be shared.
